# Vaccination Strategies Against Respiratory Pathogens in the Adult Population: A Narrative Review

**DOI:** 10.3390/vaccines14020154

**Published:** 2026-02-04

**Authors:** Laura E. Sarabia, Elizabeth Williams, Kashmira Date, Estelle Méroc, Jennifer Eeuwijk, Bradford Gessner, Joseph Bresee, Alicia Fry, Elizabeth Begier

**Affiliations:** 1 P95 Clinical and Epidemiology Services, 3000 Leuven, Belgium; estelle.meroc@p-95.com (E.M.); jennifer.eeuwijk@p-95.com (J.E.); 2 Global Medical Affairs, Pfizer Vaccines, Pfizer, Inc., New York, NY 10001, USA; elizabeth.williams@pfizer.com (E.W.); kashmira.date@pfizer.com (K.D.); brad.gessner@epivac.net (B.G.); 3 Partnership for International Vaccine Initiatives, The Task Force for Global Health, Decatur, GA 30030, USA; jbresee@taskforce.org; 4 Center for Vaccine Excellence, The Task Force for Global Health, Decatur, GA 30030, USA; afry@taskforce.org; 5 Pfizer Vaccines, Pfizer, Inc., D24 NT20 Dublin, Ireland; elizabeth.begier@pfizer.com

**Keywords:** adults, vaccination, influenza, COVID-19, pneumococcal disease

## Abstract

Respiratory infections cause substantial morbidity and mortality in older adults and other at-risk adult populations. Despite the availability of effective vaccines, adult vaccination coverage remains suboptimal. This narrative review examines strategies designed to improve vaccine uptake among non-pregnant adults aged ≥18 years and inform future adult vaccination strategies. We conducted a targeted literature search using keywords for vaccination, respiratory diseases, strategy/program/implementation, and adults in PubMed database and CDC, WHO, and ECDC websites, between 2014 and 2024. A snowball search of literature reviews and key references was also performed to identify additional relevant studies. Eligible publications focused on vaccination strategies against influenza, COVID-19, and pneumococcal disease targeting non-pregnant adults (≥18 years). We categorized the strategies by intervention type to describe their influence on vaccination campaigns and vaccine uptake/coverage. We included 45 publications, encompassing strategies focused on individual decision-making, healthcare system functions, and national policy. Educational and awareness interventions (such as healthcare worker/provider recommendations during consultation, phone calls, letters, text messages, and social media outreach) reportedly raised vaccination rates. Access-related factors, including convenient vaccination sites and free or subsidized vaccines, were reported to be important factors in improving coverage in underserved communities. Within healthcare settings, strategies such as continuous vaccine provider training and workflow/process optimization were shown to enhance vaccination delivery. At the local or national policy levels, legislation governing program targets shaped immunization efforts and facilitated collaborations and partnerships to expand campaign reach. The findings may inform policymakers and public health/immunization practitioners in designing context-specific immunization initiatives that effectively reach adult populations.

## 1. Introduction

Respiratory infections are among the leading causes of death globally [1], with older adults and other high-risk populations disproportionately affected. Over recent decades, mortality declined among children but not in older adults (≥70 years) [2]. Despite the availability of vaccines, influenza, *Streptococcus pneumoniae*, and respiratory syncytial virus (RSV) continue to drive substantial morbidity and mortality across all ages. It is estimated that seasonal influenza causes between 291,243 and 645,832 respiratory deaths worldwide each year, corresponding to 4.0–8.8 deaths per 100,000 individuals [3]. Similarly, in 2021, *S*. *pneumoniae* was associated with 97.9 million lower respiratory infections (LRIs) and 505,000 deaths [2]. As of April 2024, around 778 million COVID-19 cases and 7.1 million deaths were reported worldwide, with older adults accounting for most deaths [4]. Annually, between 2019 and 2021, RSV was estimated to cause between 4.59 and 12.5 million LRI cases and 31,500–94,900 deaths globally [2].

Vaccination remains one of the most effective tools to prevent respiratory infections, yet adult immunization rates remain low. Vaccines for pertussis, COVID-19, pneumococcus, and influenza are underutilized in adult populations [5,6]. Unlike pediatric care, adult vaccination is often not integrated into routine healthcare, and providers may not consistently recommend vaccines to their adult patients [7]. Recognizing this gap, the World Health Organization’s (WHO) Immunization Agenda 2030 established life course vaccination as a strategic priority to boost the immunization rates across age groups, including elderly populations [8], who are at higher risk of severe diseases, especially respiratory infections [9,10].

Multiple behavioral, social, and structural factors influence adult vaccination decisions. The WHO’s Behavioral and Social Drivers framework groups these influences into four domains: thinking and feeling (perceived risks, benefits, safety, and trust), social processes (social norms and recommendations), motivation (or hesitancy), and practical issues (access barriers) [11]. Limited health literacy, misinformation, and a lack of awareness about disease severity and vaccination benefits contribute to low uptake [7,12]. Logistical challenges such as transportation difficulties, inconvenient clinic hours, and out-of-pocket costs may further hinder access to vaccination services [13].

Effective adult vaccination programs require coordinated efforts across sectors. Communication strategies are essential to raise awareness and foster trust, while partnerships can expand access to convenient vaccination sites. National policies and legislation also play a critical role in shaping immunization targets and enabling program implementation [14].

This narrative review describes recent strategies used to improve adult vaccination coverage for respiratory infections, focusing on influenza, COVID-19, and pneumococcal disease. It excludes strategies targeting pregnant women, travelers, and healthcare workers (HCWs), whose motivations and barriers may differ from those of the general adult population. Previous experiences may help to develop successful strategies for adult vaccination implementation and uptake.

## 2. Materials and Methods

We conducted a narrative review of the literature published between 2014 and 2024, using a targeted search strategy across multiple sources. Peer-reviewed articles were identified through PubMed using keywords for vaccination, strategy/program/implementation, respiratory diseases, and adult populations (see Table S1 for full search terms). We also reviewed publications from the Centers for Disease Control and Prevention (CDC), WHO, and the European Centre for Disease Prevention and Control (ECDC) websites. No language restrictions were applied, and studies from all countries were considered. To supplement the primary search, we performed a snowball search of reference lists from relevant literature reviews and key articles.

Titles and abstracts were screened to identify studies describing practices influencing the success of adult vaccination campaigns. Full texts were reviewed to confirm eligibility based on reported outcomes such as vaccine uptake or impact on disease burden. Studies were included if they focused on vaccination strategies targeting non-pregnant adults (≥18 years) for influenza, COVID-19, and pneumococcal disease (caused by *S. pneumoniae*). Primary study papers that provided a detailed methodological description and reported clear results to allow for comparison were prioritized. Strategies specific to pregnant women, travelers, and healthcare workers (HCWs) were excluded due to differing behavioral drivers and intervention contexts.

Included studies were categorized by intervention type (individual level, healthcare system level, or policy level) to provide a structured overview of approaches used to improve adult vaccination coverage. This review highlights illustrative examples of successful interventions rather than attempting an exhaustive synthesis of all published literature. The selected studies were chosen to reflect a range of implementation contexts and to showcase key lessons learned from adult vaccination efforts. The statistical significance of the findings was provided when available in the studies. Given the heterogeneity in study designs and outcomes, we have conducted a qualitative synthesis to provide a nuanced understanding of each intervention’s effectiveness and implementation challenges that are crucial for policymakers to consider when adapting these strategies to their specific contexts.

## 3. Results

Vaccination rates are influenced by vaccine hesitancy, which depends on the evolving social and cultural context, and covers a wide range of attitudes, from people who are willing to be vaccinated but delay it to those who oppose vaccination [15,16]. Among the main reasons for vaccine hesitancy are complacency (e.g., perceiving a low risk of disease), convenience (e.g., affordability, availability, geographic accessibility), and trust (e.g., trust in the government, healthcare system, vaccines). Strategies to reduce these barriers to vaccination have been proven effective [15,17,18]. Vaccine hesitancy represents a set on a continuum between those who accept all vaccines to complete refusal, requiring a variety of interventions designed to reach different groups within that spectrum.

We identified 45 studies describing interventions aimed at increasing adult vaccination coverage for influenza, COVID-19, and pneumococcal disease. The most relevant outcomes were summarized by intervention type, but additional outcomes and populations may be described in the original studies. The selected studies span 18 countries: including 13 high-income, 1 upper-middle-income, 3 lower-middle-income, and 1 low-income country. Most interventions targeted influenza vaccination (n = 31 studies), followed by COVID-19 (n = 12) and pneumococcal vaccination (n = 10). The most common approaches focused on improving education and awareness (n = 14) and enhancing access to vaccination services (n = 11).

### 3.1. Individual-Level Strategies to Improve Adult Vaccination Coverage

Trust in governments and institutions is determined by social and cultural factors, and globally, is among the main factors influencing vaccination rates, particularly in low- and middle-income countries [19]. While the COVID-19 pandemic response improved preparedness for future pandemics and raised vaccination awareness, it also amplified misinformation and eroded public trust in governments, institutions, and vaccines [20]. To reduce vaccine hesitancy, institutions must regain public trust and counter misinformation [15,20]. Communication initiatives can reduce vaccine hesitancy when messages are designed for the specific context of the target population [21,22].

#### 3.1.1. Education and Awareness

##### Healthcare Provider Recommendation

Proactive vaccine recommendations by HCWs were consistently associated with increased uptake. In Shenzhen, China, influenza vaccination coverage among adults aged ≥60 years increased by 81%, and the difference in vaccine awareness was 24.7% (*p* < 0.05), following HCW recommendations during consultations [23]. Increases were also observed in Singapore [24] (from 47.7% to 80.7% among patients with chronic obstructive pulmonary disease; *p* < 0.001) and Hong Kong [25] (from 25% to 33.6% among adults ≥ 65 years; adjusted relative risk of 1.34, 95% confidence interval [CI] 1.04–1.72; *p* = 0.021) (Table 1).

##### Telephone Outreach

Telephone calls from healthcare providers or pharmacies were used to notify patients about vaccine availability or remind them to get vaccinated. In the United States (US), automated calls inviting adults ≥ 19 years to receive pneumococcal vaccines increased uptake among those who completed the call (odds ratio [OR] = 1.30, 95% CI 1.00–1.61) [26]. In Italy, vaccination coverage after physicians’ calls to splenectomized patients was 80% compared to 72% in the control group, although the difference was statistically non-significant (*p* = 0.33), most likely due to the limited sample size (n = 96 patients) [27]. In Germany, calls to liver transplant patients requesting immunization documentation led to an increase in pneumococcal vaccination rates from 46.4% to an average of 58.5% over three years [28].

##### Letters and Electronic Messaging

Letters

Mailed letters recommending vaccination improved uptake in several settings. In Ohio, US, letters sent to under-vaccinated individuals with human immunodeficiency virus increased pneumococcal vaccination coverage from 68% to 80%, which was greater compared to other medical centers without the intervention (*p* < 0.05) [29]. In Germany, printed letters to patients with chronic renal disease increased influenza vaccination rates by 8.3% when sent by physicians (*p* = 0.03) and by 3.2% when sent by health insurance funds (*p* < 0.001) [30].

Electronic letters tested in Denmark by the Nationwide Utilization of Danish Government Electronic Letter System for increasing influenza vaccine uptake (NUDGE-FLU) trials showed modest improvements. Among older adults with a history of myocardial infarction, letters emphasizing cardiovascular benefits increased influenza vaccine uptake from 82.3% to 84.9% (2.58% points; 99.55% CI −0.78–5.93). The authors report that the lack of statistical significance likely reflects the high baseline uptake, which constrained the potential for a measurable intervention effect [31]. Among adults aged 18–64 years, behaviorally designed letters increased uptake from 36.5% to 45.6% (9.1% points; 99.29% CI 7.9–10.3; *p* < 0.001) in patients with diabetes [32] and from 27.9% to 39.6% in those with other chronic conditions (11.7% points; 99.29% CI 11.2–12.2; *p* < 0.001) [33].

Text messaging

Text messages were used to promote COVID-19 and influenza vaccination. In the US, CVS Pharmacy increased COVID-19 bivalent booster uptake by 20.6% (*p* < 0.001), with the highest gains from messages encouraging appointment scheduling (23.7%, *p* < 0.001) and those reporting transmission rates (21.7%, *p* < 0.001) [34]. In Australia, text messages sent one hour before appointments in healthcare settings, combined with printed in-office reminders, increased influenza vaccination coverage from 30% to 47.2% (OR = 2.37, 95% CI 1.41–3.97) among patients with chronic conditions [35].

##### Social Media Engagement

Social media platforms were leveraged to counter misinformation and promote vaccination. In Côte d’Ivoire, a multimedia campaign increased COVID-19 vaccination coverage from 10% to 15% [37]. In Jordan, a randomized trial using Facebook Live coaching sessions led by pharmacists and physicians increased COVID-19 vaccination among hesitant adults from 0% to 51.6% (*p* < 0.002) within one month [36].

#### 3.1.2. Vaccination Access

##### Convenient Vaccination Sites

Access to vaccination sites was a key determinant of uptake (Table 2). In the US, the Minnesota Immunization Networking Initiative (MINI) deployed over 1100 vaccination sites in community settings, with convenience cited as the primary reason for vaccine acceptance [38]. In Uganda, integrating vaccination into workplaces, places of worship, and social protection payment sites increased COVID-19 coverage among adults with underlying conditions (from 6% to 11%) and those aged >50 years (from 6% to 15%) [39].

##### Workplace Vaccination Programs

Workplace vaccination initiatives improved coverage across industries. In the US, influenza vaccination rates increased among retail employees (32% to 45%, compared to workplaces without interventions) [40] and restaurant workers [41] (26% to 46%, adjusted OR = 2.33, 95% CI 1.69–3.22), when vaccines were offered during work. In Germany, companies participating in a pilot COVID-19 workplace vaccination program achieved 93.8% full vaccination coverage among employees, compared to 68% in the general population [42]. In Japan, workplace programs raised coverage from 3–4% (February-June) to 84–90% by December 2021, with lower uptake among employees of small companies [43].

##### Pharmacy-Based Vaccination

Expanding pharmacist authority to administer vaccines was associated with increased coverage. In the US, influenza vaccination rates rose by 5.5% overall and by 7.6% among adults aged 35–39 years following legislative changes [44]. In Nova Scotia, Canada, older adult vaccination rates increased from 61.8% to 73.2% over three seasons, with rural pharmacists administering a higher proportion of vaccines than their urban counterparts [45].

Although some studies found no increase in overall coverage, they reported a shift in vaccine administration from primary care to pharmacies. In Lombardy, Italy, most COVID-19 and influenza vaccines for older adults were administered in pharmacies [46]. In England and Wales, pharmacies delivered over half of influenza vaccines to adults ≥ 65 years [47,48], with coverage increasing from 30% to nearly 60% over six years in Wales [48].

The role of pharmacists in vaccination can be expanded without compromising public safety, as adverse reactions have been reported at similar rates to those in healthcare centers. Community pharmacies in Australia recorded immediate adverse events following immunization (AEFIs) in 0.05% of all COVID-19 vaccinations [49]. For influenza vaccination, 4.8% of the participants vaccinated in a pharmacy and 6% of non-pharmacy participants reported any AEFI; AEFI proportions were closer among adults aged ≥65 years (5.8% vs. 6%, respectively) [50]. Moreover, pharmacists have appropriately identified and managed AEFIs after influenza and COVID-19 vaccines [49,50,51].

#### 3.1.3. Vaccine Affordability and Monetary Incentives

##### Free Vaccination and Subsidies

Subsidized or free vaccination programs were consistently associated with increased uptake [52]. In Japan, pneumococcal vaccine coverage rose from 14.5% to 74% following a subsidy program [53,54]. In the Netherlands, offering free influenza vaccines to newly eligible adults (≥65 years) increased uptake by 9.8% (95% CI 3.5–16.1) [55]. In Ningbo City, China, free influenza vaccination at community health centers raised coverage among older adults from ~5% to 50% (OR = 11.99, 95% CI 11.87–12.11) [56] (Table 3).

In Guangdong, China, a program offering free influenza vaccines to adults ≥ 60 years included a donation and postcard-writing component. Vaccination coverage reached 60% in the intervention group (adjusted OR = 5.0, 95% CI 2.3–10.8; *p* < 0.0001) versus 20% in the standard-price group, with 96% of vaccinated individuals donating to support others’ vaccinations [57].

##### Monetary Incentives

Financial incentives modestly increased vaccine uptake. In Singapore, increasing the incentive from 10 to 20 Singaporean dollars raised influenza vaccination rates by 3% (*p* < 0.001), with greater sensitivity among individuals not in the labor force [58]. In Sweden, offering 200 Swedish kronor for COVID-19 vaccination increased uptake by 4.2% (*p* = 0.005) [59].

### 3.2. Integrated Strategies in the Healthcare System/Settings

Healthcare facilities have implemented multifaceted strategies to improve adult vaccination coverage, often combining interventions across patient engagement, provider workflow, and system-level coordination (Table 4). One example is the 4 Pillars Practice Transformation Program, which includes four evidence-based components: (1) convenient vaccination services, (2) patient communication, (3) office systems optimization, and (4) provider motivation through an immunization champion. A randomized controlled cluster trial in the US demonstrated that this program reduced missed opportunities for influenza vaccination and increased coverage by 1.44 to 5 percentage points (*p* < 0.005) [60]. Additional studies confirmed its effectiveness in improving pneumococcal and influenza vaccination rates among high-risk and older adults [61,62].

The Adult Immunization Best Practices Learning Collaboratives, implemented across US healthcare organizations, facilitated peer learning and shared tools such as immunization information systems and patient outreach strategies. Between 2014 and 2018, this approach improved pneumococcal vaccination rates from 51.0% to 59.4% and 47.8% to 56.0% in two cohorts (*p* < 0.01), and influenza vaccination rates from 37.5% to 41.1% and 38.1% to 42.6% (*p* < 0.001) [63].

A multidisciplinary intervention in internal medicine subspecialty practices, combining provider education, pre-visit counseling, and interdisciplinary huddles, resulted in an increase in pneumococcal vaccination rates from 28% to 40% over a 10-week period [64]. Similarly, a campaign in Spain targeting older adults increased influenza vaccination coverage from 56.3% to 65.8% (*p* < 0.001) through professional and public training sessions, media outreach, hospital-based recommendations, and public recognition of participating healthcare personnel [65].

The CDC-funded Partnering for Vaccine Equity initiative addressed disparities in COVID-19 vaccine uptake by training over 295,000 local trusted messengers and facilitating the administration of more than 2.1 million doses. The program improved access and addressed vaccine hesitancy among racial and ethnic minority groups, individuals with chronic conditions, disabilities, and those from lower socioeconomic backgrounds [66].

### 3.3. Policy Frameworks Supporting Adult Vaccination

National policies play a foundational role in enabling and scaling adult vaccination strategies (Table 5). In Japan, transitioning pneumococcal vaccination from a locally subsidized initiative to a national routine immunization program increased coverage from 20.9% to 40.6% and was associated with a significant reduction in mortality among elderly patients hospitalized for pneumonia (mortality trend change: −0.011; 95% CI −0.015 to −0.0064) [53,54,67].

Bhutan’s integration of influenza vaccination into routine services beginning in 2019 led to high coverage among adults aged ≥65 years, ranging from 70.9% to 84% annually between 2019 and 2022 [68]. Success factors included disease surveillance, evidence-informed planning, strong leadership, coordinated financial support, and political commitment. During the COVID-19 pandemic, Bhutan maintained influenza vaccination services through lockdowns and leveraged its infrastructure to rapidly launch a national COVID-19 vaccination program in 2021.

In Laos, the 2009 national plan for H1N1 vaccine deployment, aligned with WHO Strategic Advisory Group of Experts recommendations, provided a roadmap for pandemic response [69]. Despite initial challenges such as limited experience with seasonal influenza vaccination and concerns about safety and logistics, the campaign was successfully implemented through national oversight and responsiveness to prior avian influenza outbreaks. This experience laid the groundwork for introducing seasonal influenza vaccination through public–private partnerships.

Country-level experiences and innovations from the COVID-19 vaccination rollout have been documented by the WHO [37,70]. Key lessons include the importance of accurate population data, flexible funding mechanisms, and sustained partnerships to support lifelong immunization. These insights can inform future outbreak responses and strengthen adult vaccination programs globally.

## 4. Discussion

Improving adult vaccination coverage requires addressing a complex interplay of behavioral, logistical, and policy-level factors. Vaccine hesitancy remains a major barrier, driven by personal beliefs, misinformation, limited health literacy, and a lack of understanding of vaccine benefits [71].

While HCWs are effective messengers, particularly when recommending vaccines during consultations [23,24,25], their reach is limited to individuals actively seeking care and constrained by time and workload [72]. Additionally, HCWs may not be proactive in the recommendation of vaccines; thus, institutional support is needed for HCWs to incentivize vaccination promotion [21]. Evidence supports the effectiveness of in-person provider recommendations, but alternative communication channels such as phone calls, letters, text messages, and social media can extend reach and impact. Although these methods may yield smaller proportional increases in uptake, their scalability allows for broader population-level influence. Electronic letters or automatic vaccination reminders are low-burden and cost-effective interventions that can be implemented by healthcare providers, pharmacies, health insurers, or governmental institutions, but depend on robust information systems that may not be available in all regions [26,29,30,33,35].

The COVID-19 pandemic exacerbated hesitancy, eroding trust in previously reliable sources. To counter misinformation, interventions such as health literacy campaigns, digital tools, and community engagement have shown promise [73]. Tracking of online discussions can enable health institutions to conduct timely fact-checking of vaccine misinformation. Institutions can use online sources and social media to flag false or misleading information, disseminate vaccine education initiatives, or enhance health literacy [21]. As debunking misinformation and social media bans have had mixed results, even reinforcing false beliefs, emphasis is now being placed on proactive measures, such as providing accurate and transparent information before misinformation takes root in society or guiding the public in recognizing unreliable information [20,74]. Regret-focused reversal narratives, messages that challenge negative beliefs and highlight the consequences of non-vaccination, have been associated with increased intent to vaccinate, particularly among older adults considering RSV vaccination [75].

The success of integrated communication strategies to reduce vaccine hesitancy is influenced by both the message and the messenger. The knowledge of population characteristics and the reasons behind vaccine hesitancy, including among minority ethnic groups [34,38,74], can be used to tailor messaging based on age, health literacy, health status, risk factors, values, or beliefs, further enhancing relevance and engagement [26,27,28,29,30,31,32,33,34,35,36,37,74,76]. For example, individuals with chronic diseases have been receptive to messages designed following behavioral science concepts, which may highlight vaccine benefits for their specific condition, potential losses of not vaccinating, or prompts to action [31,32,33,34]. A non-randomized trial in Spain found that medical students exposed to online promotional campaigns were nearly 2.5 times more likely to express willingness to be vaccinated against influenza than those who received no intervention [76].

The messengers influence whether people consider the vaccine information to be reliable; depending on the social context, religious or community leaders, politicians, or HCWs could play a role in reducing vaccine hesitancy [77]. Trust in governments increased the willingness to receive the COVID-19 vaccine in Sydney and Melbourne and reduced it in New York City and Phoenix, where willingness was related to political affiliations [78]. In China, trust in physicians and the government has a greater impact on vaccine hesitancy than generalized social trust [21]. Vaccine promoters, the content of promotion messages, and the channels used to communicate with the target population should be designed for the social context and the resources available to the government and health institutions.

Prior vaccination behavior is a strong predictor of future uptake, suggesting that promoting initial vaccination can foster long-term adherence [79]. However, even motivated individuals may face logistical and financial barriers. Community-based vaccination sites, mobile teams [38,39], pharmacy-based services [44,45,46,47,48], and workplace programs [40,41,42,43] have demonstrated success in improving access, especially in rural or underserved areas. These approaches reduce scheduling burdens and travel requirements, making vaccination more convenient. Funding mechanisms such as subsidies and reimbursements can alleviate cost barriers, though eligibility criteria must be carefully designed to avoid excluding vulnerable populations [52,53,54,55,56,57].

Monetary incentives have shown mixed results [58,59]. In Germany, financial rewards up to 2000 Euros did not significantly increase COVID-19 vaccination intent among unvaccinated individuals but were effective in encouraging booster uptake among those previously vaccinated [80]. This underscores the importance of tailoring incentive strategies to the preferences and behaviors of specific target groups.

Strategies designed for one vaccine can positively influence uptake of others. For instance, pharmacy-led text message campaigns for COVID-19 vaccination also increased influenza vaccine coverage [34]. Similarly, the MINI model, developed to expand COVID-19 vaccination access, led to increased influenza vaccine distribution in underserved communities [38]. Coadministration of vaccines (e.g., RSV, influenza, and COVID-19) offers a practical and efficient approach, reducing strain on both individuals and healthcare systems [81]. Current evidence supports the safety and immunogenicity of coadministered non-live vaccines and is recommended by organizations such as the WHO and CDC [82,83].

Policy frameworks are foundational to the success of adult vaccination programs. National strategies must be supported by disease surveillance, evidence-based planning, strong leadership, coordinated funding, and political commitment. HCW recommendations are influenced by the clarity and credibility of national guidelines, which should be regularly updated to reflect evolving evidence [81]. National immunization technical advisory groups play a critical role in harmonizing recommendations and ensuring contextual relevance [8,84].

Mandatory vaccination policies, such as those targeting HCWs, have yielded mixed outcomes [85,86]. While mandates can increase coverage, they may also erode public trust and provoke resistance, as observed during the COVID-19 pandemic [37,87]. Transparent communication and community engagement are essential to mitigate these risks. Innovative tools, such as data visualization and AI-powered chatbots, can enhance benefit–risk communication [88], while peer-to-peer outreach and involvement of community leaders have proven effective in building trust [37,89].

While this review highlights diverse strategies to improve adult vaccination coverage, caution is warranted in extrapolating findings. In several studies reporting improved vaccination rates, statistical significance was either not reported or not reached, which may be due to small sample sizes, high baseline vaccination rates, and the performance of interventions. For example, the effectiveness of telephone-based reminders depends on individuals answering the call and listening to the message [26]. It is important to pinpoint that the populations considered in the selected studies varied significantly in terms of demographics, socioeconomic status, and access to healthcare. These factors could indeed influence behavioral responses to vaccination strategies. For instance, strategies that rely heavily on digital communication may be less effective in populations with limited access to technology. The elderly may face mobility issues and require more accessible vaccination locations, such as home visits or community centers. Conversely, working adults may have better access to healthcare facilities due to employment benefits. Also, community-based approaches tend to be more successful in settings with strong local networks and trust in community leaders. Understanding these differences is crucial for tailoring interventions to specific settings to better meet the needs of each group, ensuring higher coverage and effectiveness [90,91,92]. Moreover, most studies in this review were conducted in high-income countries, and their applicability may be limited in settings with different healthcare infrastructure, socioeconomic conditions, and cultural contexts. For example, interventions that rely on advanced medical facilities or extensive public health campaigns may not be feasible in low-income countries with limited resources. The COVID-19 pandemic illustrated how vaccine access was constrained by financial limitations, export restrictions, and cold-chain requirements for mRNA vaccines in low-income countries [37]. Additionally, cultural beliefs and misinformation often play a significant role in vaccine acceptance in low-income settings. For instance, a review highlighted how community engagement and mobile vaccination units were effective in increasing vaccination rates in rural areas of sub-Saharan Africa [93].

Additionally, differences in disease burden, vaccine familiarity, and seasonal variability can influence uptake [15,94,95]. For example, influenza vaccines may be more readily accepted due to longstanding awareness, while newer vaccines like those for COVID-19 may initially generate higher demand due to heightened concern [94]. These contextual factors must be considered when adapting strategies to new settings or antigens.

## 5. Conclusions

This review presents practical, evidence-based strategies to overcome barriers to adult vaccination against respiratory infections. By targeting multiple vaccines through each intervention and integrating tailored communication, accessible delivery models, and supportive policies, decision makers can strengthen adult immunization programs and advance public health resilience.

## Figures and Tables

**Table 1:** Summary of adult vaccination strategies by communication channel.

Reference	Country/Location	Target Population	Study Period	Vaccine	Strategy Category	Strategy Type (Level)	Outcome	Implementer	Notes/Limitations
HCW Education and Face-to-Face Recommendations									
You et al. 2023 [23]	China	Adults ≥ 60 years	2016–2018	Influenza	Provider-led education	In-person recommendation (Local)	+81% vaccinated (from 1357 to 2457)	Community health center	Strong increase; limited to clinic visitors
Li et al. 2019 [24]	Singapore	COPD patients	2014	Influenza	Provider-led education	Posters + HCW recommendation (Local)	Uptake increased from 47.7% to 80.7%; 87.9% after physician recommendation	National University Hospital	High impact; single center
Leung et al. 2017 [25]	Hong Kong	Adults ≥ 65 years	2015	Influenza	Peer education	Medical student presentations (Local)	Uptake: 33.6% vs. 25% control (+8.6 pp)	Hong Kong Hospital Authority	Short intervention; limited scalability
Telephone-Based Reminders									
Stolpe & Choudhry 2019 [26]	US	Adults ≥ 19 years	2016–2017	Pneumococcal	Automated outreach	Pharmacy robocalls (Local)	OR = 1.30 for call completers; overall rates ~2%	Pharmacy chains (NY, PA, VT)	Low baseline coverage; modest effect
Bianchi et al. 2019 [27]	Italy	Splenectomized patients	2014–2017	Influenza	Provider-led outreach	Physician phone call (Local)	Coverage: 80% vs. 72% control (+8 pp)	Bari Policlinico Hospital	High baseline coverage
Dehnen et al. 2019 [28]	Germany	Liver transplant patients	2014–2018	Influenza/Pneumococcal	Provider-led outreach	Phone call reminder to bring records (Local)	Influenza: 28.9% (2016) vs. 11.5–19% (2010–2012); Pneumococcal: 60.1% (2016) vs. 46.4% (2013)	University Hospital Essen	Longitudinal data; no control group
Written Communications (Letters)									
Burns et al. 2018 [29]	US	Veterans with HIV	2015	Pneumococcal	Provider-led outreach	Letter + standing order (Local)	Coverage increased from 68% to 80%; 38% vaccinated within 180 days	Veterans Affairs Medical Center	Targeted population; high baseline
Schulte et al. 2019 [30]	Germany	Renal disease patients	2012–2017	Influenza	Mixed sender outreach	Physician letter (Local)	+8.3 pp vs. control	German Association of Statutory Health Insurance Physicians	Sender credibility matters
Bhatt et al. 2024 [31]	Denmark	Adults ≥ 65 with CVD	2022–2023	Influenza	Government communication	Electronic letter on CVD benefits (National)	Uptake (patients with acute myocardial infarction): 84.9% vs. 82.3% usual care (+2.6 pp)	NUDGE-FLU trial	High baseline; small gain
Lassen et al. 2024 [32]	Denmark	Adults 18–64 years with chronic disease	2023–2024	Influenza	Government communication	Electronic invitation letter (National)	Uptake: 45.6% vs. 36.5% usual care (+9.1 pp)	NUDGE-FLU trial	Focused on diabetes
Johansen et al. 2024 [33]	Denmark	Adults 18–64 years with chronic disease	2023–2024	Influenza	Government communication	Electronic invitation letter (National)	Uptake: 39.6% vs. 27.9% usual care (+11.7 pp)	NUDGE-FLU-CHRONIC trial	Strong effect; chronic disease focus
Text Message Reminders									
Milkman et al. 2024 [34]	US	CVS adult patients	2022	COVID-19	Automated outreach	SMS booster reminders (Local)	+20.63% booster uptake after 30 days	CVS Pharmacy	High scalability
Gonzalez-Chica et al. 2024 [35]	Australia	Adults 18–64 years with chronic conditions	2020–2021	Influenza	Automated outreach	SMS 1 h before appointment + printed reminder (National)	Uptake: 47.2% vs. 30% control (+17.2 pp)	General practices	Timing and layering matter
Social Media and Digital Outreach									
Abdel-Qader et al. 2022 [36]	Jordan	Adults 18–64 years	2021	COVID-19	Digital engagement	Facebook Live coaching (Local)	Uptake: 0% to 51.6% vs. 0% to 5.36% control	Pharmacists and physicians	Strong effect; small sample

COPD: chronic obstructive pulmonary disease; CVD: cardiovascular disease; HCW: healthcare worker; HIV: human immunodeficiency virus; NUDGE: Nationwide Utilization of Danish Government Electronic Letter System; OR: odds ratio; pp: percentage point; US: United States.

**Table 2:** Summary of adult vaccination strategies by vaccination sites.

Reference	Country/Location	Targeted Population	Study Period	Vaccine	Strategy Category	Strategy Type (Level)	Outcomes	Implementers	Notes/Limitations
Vaccination Sites and Workplace Vaccination									
Johansen et al. 2024 [38]	US	Underserved communities	2021–2023	COVID-19/Influenza	Convenient vaccination sites	Infrastructure and vaccination teams (Local)	From 100–125 to over 400 sites provided from 5700–7600 to 19,500 COVID-19 and 7250 influenza vaccines annually	Minnesota Immunization Networking Initiative	Refugee, immigrant, and migrant communities
Kiiza et al. 2024 [39]	Uganda	Priority populations for vaccination	2021	COVID-19	Convenient vaccination sites	Vaccine deployment targeting vulnerable groups (Local)	Uptake increase: 6–15% in adults > 50 years	Makerere University	Targeted population
Montejo et al. 2017 [40]	US	Retail employees ≥ 18 years	2015–2016	Influenza	Convenient vaccination sites	Workplace vaccination (Local)	Coverage: 45% vs. 32% (site without intervention)	Two retail locations with on-site clinics	Short intervention
Graves et al. 2016 [41]	US	Restaurant employees ≥ 18 years	2011–2012	Influenza	Convenient vaccination sites	Workplace vaccination (Local)	Coverage increased from 26% to 46%	Restaurants in the Seattle area	Employers spent < 3 h on the intervention
Wagner et al. 2023 [42]	Germany	Employees from diverse industries	2021–2022	COVID-19	Convenient vaccination sites	Workplace vaccination (Local)	57.7% employees vaccinated in the workplace	Companies in Baden-Württemberg	High operational burden
Mori et al. 2022 [43]	Japan	Employees in Japan	2020–2021	COVID-19	Convenient vaccination sites	Workplace vaccination (National)	Coverage: from 3–4% in February to 84–90% in December	Companies in Japan	Lower uptake among small companies
Pharmacists									
Drozd et al. 2017 [44]	US	Adults ≥ 18 years	2003–2013	Influenza	Pharmacy-based vaccination	Regulation changes (National)	Uptake: 7.6% increase among 35–39-year-olds	Pharmacies	Self-reported survey data
Isenor et al. 2018 [45]	Canada	Adults ≥ 65 years	2006–2016	Influenza	Pharmacy-based vaccination	Publicly funded program + pharmacy vaccination (Local)	Coverage from 61.8% (2012/13) to 71.6% (2013/14)	Nova Scotia Department of Health and Wellness	Secondary data from health claims
Pennisi et al. 2024 [46]	Italy	Adults ≥ 18 years	2021–2023	COVID-19/Influenza	Pharmacy-based vaccination	Regulation changes (Local)	Administration of 46% of COVID-19 and 17% of influenza vaccines	Ministry of Health and the Lombardy Region	Reach vulnerable populations
Rai and Wood 2018 [47]	UK	Adults ≥ 65 and 18–64 years in a risk group	2013–2015	Influenza	Pharmacy-based vaccination	Vaccination service for 2014/15 season (Local)	8743 vaccines administered, mainly to older adults	Pharmacies in West Midlands	No effect on uptake rates; high patient satisfaction
Deslandes et al. 2020 [48]	UK	Adults ≥ 18 years	2012–2018	Influenza	Pharmacy-based vaccination	Community pharmacies (Local)	103,941 vaccines administered	Pharmacies in Wales	No effect on vaccine uptake; older adults the main recipients

UK: United Kingdom; US: United States.

**Table 3:** Summary of adult vaccination strategies for vaccine affordability and monetary incentives.

Reference	Country/Location	Targeted Population	Study Period	Vaccine	Strategy Category	Strategy Type (Level)	Outcomes	Implementers	Notes/Limitations
Free Vaccination/Subsidies									
Shono et al. 2018 [53]	Japan	Adults ≥ 65 years	2015	Pneumococcal	Vaccine subsidy	Age eligibility in 5-year increments (National)	Coverage increased from 14.5% to 33.5% after 1 year	Routine vaccination program	Web-based survey of self-selected population
Naito et al. 2020 [54]	Japan	Adults ≥ 65 years	2014–2018	Pneumococcal	Vaccine subsidy	Age eligibility in 5-year increments (National)	Coverage reached 74%; annual rate increased from 2–5% to 10–11%	Routine vaccination program	Survey of vaccines shipped by providers
Van Ourti and Bouckaert 2020 [55]	The Netherlands	Adults ≥ 65 years	1996–2008	Influenza	Free vaccination	Invitation for every Dutch inhabitant > 65 years (National)	+9.8 pp in vaccine uptake	Dutch vaccination program	Small impact on hospitalizations and deaths
Ye et al. 2024 [56]	China	Adults ≥ 60 years	2017–2023	Influenza	Free vaccination	Expansion of eligible ages (Local)	Coverage increased from ~5% to 50.05%	Vaccination clinics in Ningbo City	Effects of early COVID-19 pandemic
Mc Hugh et al. 2015 [52]	Ireland	Adults ≥ 50 years with medical cards	2009–2011	Influenza	Vaccine reimbursement	Free vaccination for medical card holders with income-based eligibility (National)	Coverage of medical card holders was higher than in those with private insurance only (78.7% vs. 59.0%)	Irish Longitudinal Study on Ageing	No information on the timing of eligibility and vaccination
Wu et al. 2022 [57]	China	Adults ≥ 60 years	2020–2021	Influenza	Pay-it-forward (free vaccination)	Voluntary donations for subsequent vaccinations (Local)	Uptake increased from 20% to 60%	Clinics in Guangdong province	Potential Hawthorne effect; community engagement
Monetary Incentives									
Yue et al. 2020 [58]	Singapore	Adults ≥ 65 years	2018	Influenza	Monetary incentive	Shopping vouchers of 10–30 Singaporean dollars (National)	Uptake increased from 4.5% to 7.5%, increasing the incentive from 10 to 20 Singaporean dollars	Singapore Population Health Study	Low participation rate
Campos-Mercade et al. 2021 [59]	Sweden	Adults 18–49 years	2021	COVID-19	Monetary incentive	200 Swedish kronor (National)	4.2% points increase (from 71.6% control) in vaccine uptake	General sample of the Swedish population	Only one amount of incentive tested

pp: percentage point.

**Table 4:** Summary of adult vaccination strategies for healthcare system.

Reference	Country/Location	Targeted Population	Study Period	Vaccine	Strategy Category	Strategy Type (Level)	Outcomes	Implementers	Notes/Limitations
Lin et al. 2016 [60]	US	Adults ≥ 18 years	2012–2015	Influenza	Multifaceted strategy	4 Pillars Toolkit (Local)	Uptake increased from 53.7% to 58.7% (Pittsburgh) and from 36.7% to 39.4% (Houston)	Clinical practices in Pittsburgh and Houston	Potential Hawthorne effect; late vaccine delivery in year 1
Nowalk et al. 2017 [61]	US	Adults 18–64 years in a risk group	2012–2015	Influenza/Pneumococcal	Multifaceted strategy	4 Pillars Toolkit (Local)	Coverage increased from 43.5% to 55.7% for pneumococcal and from 52.1% to 56.8% for influenza	Clinical practices in Pittsburgh and Houston	ICD-9 coding not verified; limited study population
Zimmerman et al. 2017 [62]	US	Adults ≥ 65 years	2012–2015	Pneumococcal	Multifaceted strategy	4 Pillars Toolkit (Local)	Increase in PPV and PCV rates: HR (95% CI) PPV: 1.78 (1.20–2.63) and PCV: 14.69 (6.46–33.39)	Clinical practices in Pittsburgh and Houston	Potential Hawthorne effect
Ciemins 2020 et al. [63]	US	Adults ≥ 18 years	2017–2018	Influenza/Pneumococcal	Multifaceted strategy	Scale-up Learning Collaborative Approach (Local)	Increase in pneumococcal vaccine uptake in ≥65 years in two cohorts: from 51.0% to 59.4% and from 47.8% to 56.0%. Increase in influenza vaccine uptake in one cohort: from 38.1% to 42.6%	Adult Immunization Best Practices Learning Collaboratives	Limited control providers available; race/ethnic disparities not accounted for
Shafer et al. 2021 [64]	US	Adults 18–64 years	NA	Pneumococcal	Multifaceted strategy	Clinician education webinar, pre-visit counseling phone call, and pre-visit interdisciplinary team (Local)	Increase in vaccination rate from 28% to 40%	Internal medicine subspecialty practices	Short intervention phase; 38% of the eligible population was not reached
Fernández-Prada et al. 2021 [65]	Spain	Adults ≥ 65 years	2019–2020	Influenza	Multifaceted strategy	Training sessions, information dissemination through the media, recommendations for vaccination, social recognition (Local)	Vaccination rate increased from 56.3% to 65.8%	HCW and general public	Strategy designed for the specific study area/population
Fiebelkorn et al. 2024 [66]	US	Disproportionately affected adult populations	2021–2022	COVID-19/Influenza	Multifaceted strategy	Partnering for Vaccine Equity Program (National)	295,000 trusted messengers trained; 2.1 million COVID-19 vaccinations	Partners funded to address vaccine equity	Due to non-research partnerships, the effectiveness of interventions was not determined

CI: confidence interval; HCW: healthcare worker; HR: hazard ratio; NA: not applicable; PCV: pneumococcal conjugate vaccine; PPV: pneumococcal polysaccharide vaccine; US: United States.

**Table 5:** Summary of policy framework for adult vaccination strategies.

Reference	Country/Location	Targeted Population	Study Period	Vaccine	Strategy Category	Strategy Type (Level)	Outcomes	Implementers	Notes/Limitations
Kobayashi et al. 2021 [67]	Japan	Adults ≥ 65 years	2011–2017	Pneumococcal	National immunization program	Routine immunization program (National)	Vaccine rate increased from ~20% to >40%	Japan’s national PPSV23 vaccination program (Japanese Diagnosis Procedure Combination database)	Potential inclusion of hospital-acquired pneumonia cases; no confirmation of pneumococcal pneumonia; no time or confounder adjustments
Wangchuk et al. 2023 [68]	Bhutan	Adults ≥ 65 years, high-risk	2019–2022	Influenza	National immunization program	Bhutan’s routine immunization services (National)	Yearly coverage rates in adults ≥ 65 years; range: 70.9–84%	National Technical Advisory Group, Ministry of Health	No centralization of coverage data
Xeuatvongsa et al. 2015 [69]	Laos	Laos population	2009–2010	Influenza	Vaccine deployment	Pandemic influenza vaccine deployment (National)	14% vaccine coverage in the Lao population	National Emerging Infectious Disease Coordinating Office	Limited sample and no true prevalence estimates
Palmer et al. 2022 [70]	Global	Global population	Up to May 2021	COVID-19	Vaccine deployment	Pandemic COVID-19 vaccine deployment (International)	Lessons learned from vaccine roll-outs and the perspectives of participants in the process	Small Countries Initiative, WHO European Centre for Investment for Health and Development	Limited to the initial rollout phase
WHO 2023 [37]	Global	Adults and people living with comorbidities	2020–2023	COVID-19	Vaccine deployment	Pandemic COVID-19 vaccine deployment (International)	Lessons learned: preparedness for future pandemics, improvement of components of immunization programs, and primary health care services	Global and regional agencies, country decision makers, program managers, and development partners who support national immunization programs	Limited global vaccine supply in the initial months; delays in vaccine shipments complicated the scheduling of vaccination sessions

PPSV23: 23-valent pneumococcal polysaccharide vaccine; WHO: World Health Organization.

## Data Availability

No new data were created or analyzed in this study. Data sharing is not applicable to this article.

## Supplementary Materials

The following supporting information can be downloaded at: https://www.mdpi.com/article/10.3390/vaccines14020154/s1, Table S1: Search strategy.

## Abbreviations

The following abbreviations are used in this manuscript:

AEFI	adverse event following immunization
CDC	Centers for Disease Control and Prevention
CI	confidence interval
COPD	chronic obstructive pulmonary disease
CVD	cardiovascular disease
ECDC	European Centre for Disease Prevention and Control
HCW	healthcare worker
HIV	human immunodeficiency virus
HR	hazard ratio
LRI	lower respiratory infection
MINI	Minnesota Immunization Networking Initiative
NA	not applicable
NUDGE	Nationwide Utilization of Danish Government Electronic Letter System
OR	odds ratio
PCV	pneumococcal conjugate vaccine
pp	percentage points
PPV	pneumococcal polysaccharide vaccine
RSV	respiratory syncytial virus
WHO	World Health Organization
