# Supplementary material for: Vaccination Strategies Against Respiratory Pathogens in the Adult Population: A Narrative Review
Source: Vaccines (Basel). 2026 Feb 4;14(2):154. doi: 10.3390/vaccines14020154 (PMC12945287; doi:10.3390/vaccines14020154)
Supplement: Supplementary file 1 [file vaccines-14-00154-s001.zip › vaccines-4078734-supplementary.pdf]

Table S1. Search strategy

Database	Search string	Hits
Medline (PubMed)	((("Vaccination"[Mesh] OR "vaccin*"[Title/Abstract] OR "vaccination*"[Title/Abstract] OR "immuniz*"[Title/Abstract] OR "immunis*"[Title/Abstract] OR "Immunization Programs"[Mesh] OR "Immunization Program*"[Title/Abstract] OR "vaccination program*"[Title/Abstract] OR "vaccination campaign"[Title/Abstract]) AND ("strateg*"[Title/Abstract] OR "implementation"[Title/Abstract] OR "polic*"[Title/Abstract] OR "organis*"[Title/Abstract] OR "organiz*"[Title/Abstract] OR "program*"[Title/Abstract] OR "best practice*"[Title/Abstract])) AND (("B. pertussis"[Title/Abstract] OR "Whooping Cough"[MeSH Terms] OR "Whooping Cough"[Title/Abstract] OR "pertussis"[Title/Abstract] OR "Bordetella pertussis"[MeSH Terms]) OR (("SARS-CoV-2"[Title/Abstract] OR "COVID-19"[MeSH Terms] OR "COVID-19"[Title/Abstract] OR "SARS-CoV-2"[MeSH Terms] OR "SARS-CoV-2"[Title/Abstract] OR "novel coronavirus"[Title/Abstract]) AND (meta-analysis[Filter] OR review[Filter] OR systematicreview[Filter])) OR (Influenza*[Title/Abstract] OR "Human Flu"[Title/Abstract] OR flu[Title/Abstract] OR flue[Title/Abstract] OR "Influenza, Human"[Mesh]) OR ("Streptococcus pneumoni*"[Title/Abstract] OR "Diplococcus pneumoni*"[Title/Abstract] OR "Pneumococc*"[Title/Abstract] OR "S pneumoni*"[Title/Abstract]) OR ("respiratory syncytial"[Title/Abstract] OR "syncytial virus"[Title/Abstract]) OR ("Mycobacterium tuberculosis"[Title/Abstract] OR "Tuberculosis"[Title/Abstract] OR "Tuberculosis"[Mesh]))) NOT (Animals[Mesh] NOT (Humans[Mesh] AND Animals[Mesh])) AND (2014:2024[pdat]) AND (alladult[Filter] OR adult[Filter] OR middleagedaged[Filter] OR middleaged[Filter] OR aged[Filter] OR 80andover[Filter] OR youngadult[Filter])<br><br>**Search date: 23/10/2024**	**3,657**
